# Enhanced Metabolic Control in a Pediatric Population with Type 1 Diabetes Mellitus Using Hybrid Closed-Loop and Predictive Low-Glucose Suspend Insulin Pump Treatments

**DOI:** 10.3390/pediatric16040100

**Published:** 2024-12-14

**Authors:** Irina Bojoga, Sorin Ioacara, Elisabeta Malinici, Victor Chiper, Olivia Georgescu, Anca Elena Sirbu, Simona Fica

**Affiliations:** 1 Department of Endocrinology, Diabetes Mellitus, Nutrition and Metabolic Disorders, “Carol Davila” University of Medicine and Pharmacy, 050474 Bucharest, Romania; irina.bojoga@drd.umfcd.ro (I.B.);drelisabetasava@yahoo.com (E.M.); victor-george.chiper@drd.umfcd.ro (V.C.); olivia.georgescu@umfcd.ro (O.G.); anca.sirbu@umfcd.ro (A.E.S.); simona.fica@umfcd.ro (S.F.); 2 Department of Endocrinology, Diabetes Mellitus, Nutrition and Metabolic Disorders, “Elias” University Emergency Hospital, 011461 Bucharest, Romania

**Keywords:** hybrid closed-loop systems, automated insulin delivery systems, insulin pump, pediatric diabetes, type 1 diabetes, glycemic control, technological devices

## Abstract

Background: Insulin pumps coupled with continuous glucose monitoring sensors use algorithms to analyze real-time blood glucose levels. This allows for the suspension of insulin administration before hypoglycemic thresholds are reached or for adaptive tuning in hybrid closed-loop systems. This longitudinal retrospective study aims to analyze real-world glycemic outcomes in a pediatric population transitioning to such devices. Methods: We evaluated children with type 1 diabetes mellitus (T1D) admitted to the Pediatric Diabetes Department from a major University Hospital in Bucharest, Romania, who transitioned to hybrid closed-loop or predictive low-glucose suspend system from either non-automated insulin pumps or multiple daily injections. The primary outcome was assessing the change in glycated hemoglobin (HbA1c) after initiating these devices. Secondary outcomes analyzed changes in glucose metrics from the 90 days prior to the baseline and follow-up visit. Results: 51 children were included (58.8% girls), the mean age was 10.3 ± 3.7 years, and the mean follow-up duration was 13.2 ± 4.5 months. The analyzed parameters, such as HbA1c (6.9 ± 0.7% vs. 6.7 ± 0.6%, *p* = 0.023), time in range (69.3 ± 11.2% vs. 76 ± 9.9%, *p* < 0.001), time in tight range (47.4 ± 10.9% vs. 53.7 ± 10.7%, *p* < 0.001), time below range (5.6 ± 2.9% vs. 3.5 ± 1.9%, *p* < 0.001), time above range (25 ± 11.2% vs. 20.4 ± 9.4%, *p* = 0.001), and coefficient of variation (37.9 ± 4.8% vs. 35.6 ± 4.6%, *p* = 0.001), showed significant improvements. Conclusions: The application of these sensor-integrated insulin pumps can significantly enhance metabolic control in pediatric populations, minimizing glycemic variations to mitigate complications and enrich the quality of life.

## 1. Introduction

Type 1 diabetes mellitus (T1D) is one of the most frequent endocrine pathologies in children and its prevalence is continuously rising [1]. The treatment of this chronic pathology may represent a burden for children and their guardians [2]. In recent years, the management of T1D has dramatically evolved due to technological advancements aimed at enhancing metabolic control and the quality of life for individuals and their caregivers. Significant developments include more precise and extended-use continuous glucose monitoring systems (CGMSs) and increasingly sophisticated insulin delivery devices [3]. First, glycemic control was improved when individuals started to use CGMSs and insulin pumps as standalone devices. After that, the first step towards obtaining a fully closed-loop system was connecting the two devices and creating an algorithm that suspends the basal rate delivery when the CGMS measurement detects hypoglycemia. This system, known as low glucose suspend (LGS), is used by the MiniMed Paradigm Veo and MiniMed 530G devices. Basal rate delivery resumes after 2 h unless the user manually resumes it earlier. More recent innovations include predictive low glucose suspend (PLGS) insulin pumps. These advanced devices can proactively stop insulin delivery to prevent impending hypoglycemia [4]. The basal insulin delivery resumes when sensor glucose recovers. PLGS systems are represented by Medtronic MiniMed 640G and MiniMed 740G. These two insulin pumps share the same therapy algorithm, the difference between them being that MiniMed 740G features an in-built Bluetooth technology that allows the pump to connect to compatible mobile devices and upload data to Carelink Software System directly, without the need for an adapter.

Hybrid closed-loop (HCL) systems represent the latest frontier, integrating a CGM sensor, a data-analyzing algorithm, and an insulin pump that variably adjusts insulin doses based on CGM readings [5]. The user is still required to count the number of carbohydrates and administer the meal bolus accordingly, meaning the system is not fully automated. Currently, these systems are single-hormone, supplying insulin only; however, dual-hormone closed-loop systems are being tested in randomized clinical trials [6]. These new technologies aim to enhance the quality of life and assist individuals with T1D in achieving better metabolic control while preventing hypoglycemia. The currently available hybrid closed-loop systems are the Medtronic 670G/780G, Tandem t:slim X2 and Mobi with Control IQ technology, Omnipod 5, Diabeloop, and CamAPS FX systems [7,8]. Each device has specific variables needed to initiate the pump, user-modifiable parameters, and fixed parameters that cannot be changed by the user. These aspects can vary from one system to another.

The Medtronic MiniMed 670G was the first HCL system commercially available. It can function in two modes: manual mode, as a traditional insulin pump, or auto mode, when paired with Guardian 3 CGM. The algorithm adjusts basal insulin delivery every five minutes in auto mode based on CGM readings, targeting a 120 mg/dL glucose level. The Medtronic 780G system utilizes Guardian 4 sensors and a transmitter, and it can also operate in both manual and auto modes. Compared to the MiniMed 670G system, the 780G features a redesigned algorithm with modifications to the closed-loop exit conditions, which reduces user burden while ensuring safety. Alongside basal insulin adjustments, this system can provide a correction bolus every 5 min, if needed, based on sensor glucose readings [9]. It also detects the rapid rise of glucose, predicting that the person had an unannounced meal (meal detection technology). It can be used with extended-use infusion sets that can be worn for up to 7 days. During auto mode, the only parameters that need to be adjusted are the insulin-to-carbohydrate ratio (I:C ratio), active insulin time, and glucose target (as low as 100 mg/dL). All improvements from the previous HCL generation aim to assist people in achieving better glycemic control and improving their quality of life. This HCL system is approved to be used by individuals with T1D aged seven and older [5,10].

The T:slim X2 insulin pump uses Control-IQ technology and can be paired with a compatible CGM to create an automated insulin device (AID). The adjustable settings include I:C ratio, basal rate, and correction factor, along with a selectable sleep/activity mode. It is approved for people aged six and older. The new version, Tandem Mobi, is half the size of T:slim X2 and may be controlled by a smartphone [11].

Omnipod 5 is a tube-free insulin pump comprised of the Pod (a small, disposable device, that the user fills with insulin and wears directly on his body) and a remote controller, compatible with Dexcom G6, Dexcom G7, and FreeStyle Libre 2 Plus CGMS. The system can also be controlled with the Omnipod 5 App installed on a compatible smartphone. It has a customizable target glucose and I:C ratio and the correction factor can also be modified by the user. It is approved to be used for people aged two and older [12].

Diabeloop AID features the Kaleido or AccuChek Insight insulin pump, the Dexcom G6 CGM, and an Android controller. It has two variants: Diabeloop Generation 1 and Diabeloop for Highly Unstable Type 1 Diabetes [13].

CamAPSFx system is an Android application compatible with Dexcom G6 and Libre 3 as CGMS devices and with Ypsomed Ypsopump, Dana Diabecare RS, and Dana-I as insulin pumps. It requires a compatible smartphone and is suitable for individuals aged one and older. Notably, it is currently the only HCL system approved for use during pregnancy [7].

Since 2013, individuals with T1D and their caregivers have taken the initiative, utilizing open-source software to develop and enhance their own hybrid closed-loop systems [14]. Until recently, “do-it-yourself” systems for managing diabetes were unregulated and lacked medical approval, despite being used by many individuals. In 2023, Tideloop became the first and only “do-it-yourself” HCL system to receive FDA approval for people with diabetes aged six and older.

In 2023, FDA also cleared the iLet Bionics system [15]. Unlike the HCL systems mentioned earlier, the bionic pancreas does not require carbohydrate counting, makes all dosage decisions automatically, and is initialized based on body weight [16]. It is compatible with Dexcom G6 and G7 CGMS.

At the moment, the only PLGS and HCL systems available in our country are Medtronic MiniMed 640G, 740G, and 780G, so these are the devices used by individuals in this current study.

Glycated hemoglobin (HbA1c) reflects the average glycemia over the past 90 days and is the main parameter for assessing glycemic control, having a strong predictive value for future diabetes complications. The American Diabetes Association (ADA) and the International Society for Pediatric and Adolescent Diabetes (ISPAD) recommend a target HbA1c < 7% for most children and adolescents [17,18], with a lower target of 6.5% for those using CGMSs or automated insulin delivery systems [18]. However, HbA1c has some limitations: it doesn’t capture glycemic variability, or the frequency of hypoglycemia, and may be unreliable in certain conditions (for example, hemoglobin abnormalities) [19]. Additional metrics from CGM, such as time in range (TIR), time in tight range (TITR), time below range (TBR), time above range (TAR), and coefficient of variation (CV), can provide further insights into glycemic control. The International Consensus on Use of Continuous Glucose Monitoring defines stable glucose levels as CV < 36% [20]. The ADA Standards of Care and the International Consensus Report on TIR recommend that individuals with diabetes should aim to spend at least 70% of the day within the glycemic target range of 70 to 180 mg/dL, less than 4% of their time below 70 mg/dL, less than 1% below 54 mg/dL, less than 25% above 180 mg/dL, and less than 5% above 250 mg/dL [21,22,23]. Many children and adolescents with T1D do not meet recommended targets, highlighting the need for innovative therapeutic approaches. CGMSs provide numerous interstitial glucose readings, but they can sometimes lack accuracy [19]. Therefore, healthcare practitioners and users should not rely solely on CGM metrics. For a thorough assessment of glycemic control, HbA1c levels should be corroborated with data from CGMS devices.

The primary objective of this study was to evaluate the change in HbA1c levels after initiating HCL or PLGS insulin therapy in children with T1D. The secondary objectives included measuring changes in TIR, defined as glucose levels between 70 and 180 mg/dL, and TITR, defined as 70–140 mg/dL. We also assessed changes in TAR at various thresholds: above 180 mg/dL, between 180–250 mg/dL, and above 250 mg/dL. TBR was evaluated for levels below 70 mg/dL, within the range of 54–70 mg/dL, and below 54 mg/dL. Additionally, we measured the changes in the CV. Furthermore, the children were screened for diabetic complications, and assessments for associated autoimmune diseases were conducted according to established guidelines.

## 2. Materials and Methods

### 2.1. Study Design and Participants

We conducted a longitudinal retrospective study on children with T1D transitioning from either non-automated insulin pumps or multiple daily injections (MDI) to PLGS or HCL insulin pump therapy (baseline visit) between 2019 and 2024. All participants were admitted to the Pediatric Diabetes Department of a major University Hospital in Bucharest, Romania. Informed consent was obtained from all legal guardians of individuals included in this study. The research has complied with all the relevant national regulations and institutional policies, following the tenets of the Helsinki Declaration, and has been approved by the local ethics committee. The inclusion criteria were an age of less than 18 years old at admission and T1D diagnosis. At baseline, we excluded individuals with diabetes duration under 6 months, or not already using a CGM, or with other types of diabetes mellitus, such as MODY, neonatal diabetes, and type 2 diabetes. The follow-up visit was the one closest to 12 months after baseline. The minimum follow-up period was 6 months. All analyzed data were obtained from an anonymized database that did not contain personally identifiable information.

### 2.2. Clinical and Paraclinical Investigations

For each subject, the patient form was completed with information regarding family and personal history, the duration of diabetes, and details related to diabetes treatment and management. This included the use of CGMS, insulin pumps, total daily insulin dosage, and the calculation of insulin dosage per kilogram of body weight over 24 h. A physical examination was conducted, including anthropometric measurements, pubertal stage assessment, and blood pressure evaluation. The body mass index (BMI) was calculated (body weight [kg]/body height [m]^2^), and percentiles were determined. Participants were also asked about the presence of symptomatology suggestive of diabetic neuropathy. In addition to measuring HbA1c, other laboratory tests were performed: total cholesterol, LDL cholesterol, HDL cholesterol, triglycerides, aspartate aminotransferase (AST), and alanine transaminase (ALT). Considering the frequent association between T1D and other autoimmune pathologies, particularly thyroid diseases, thyroid function was assessed by measuring thyroid stimulating hormone (TSH) and free thyroxine (fT4). To screen for Hashimoto’s disease, we measured the levels of anti-thyroid peroxidase (TPO) antibodies and anti-thyroglobulin (Tg) antibodies. Additionally, in children who were not already diagnosed with celiac disease and following a gluten-free diet, tissue transglutaminase IgA (tTg-IgA) levels were checked to screen for this pathology. Urine albumin–creatinine ratio (uACR) testing and an ophthalmological consultation, including a fundoscopic examination, were conducted to screen for microvascular complications. This screening for complications and related pathologies followed the guidelines set forth by the ADA [24]. During follow-up visits, to assess the safety of using the PLGS and HCL systems, participants were asked about any episodes of severe hypoglycemia, instances of diabetic ketoacidosis (DKA), and any unscheduled hospital admissions due to hyperglycemia or hypoglycemia.

HbA1c was measured using high-performance liquid chromatography (HPLC). Total cholesterol, HDL, LDL, triglycerides, AST, and ALT levels were evaluated with spectrophotometric methods. TSH and free T4 levels were measured via electrochemiluminescence immunoassay. Anti-TPO, anti-Tg, and tTG-IgA antibodies were assessed using the ELISA method. A spot urine specimen was collected for the uACR, calculated through immunoturbidimetry. Diabetic retinopathy screening included a fundoscopic eye examination by an ophthalmologist.

HbA1c and CGM metrics were assessed and compared at baseline and during follow-up visits. The CGM metrics evaluated included the following: TIR (the percentage of readings and time spent within the range of 70–180 mg/dL), TITR (the percentage of readings and time spent within the range of 70–140 mg/dL), TBR < 70 mg/dL (the percentage of readings and time spent below 70 mg/dL), TBR 54–70 mg/dL (level 1 hypoglycemia, measured as the percentage of readings and time spent in this range), TBR < 54 mg/dL (level 2 hypoglycemia, measured as the percentage of readings and time spent below this level), TAR >180 mg/dL (the percentage of readings and time spent above 180 mg/dL), TAR 180–250 mg/dL (level 1 hyperglycemia, measured as the percentage of readings and time spent in this range), TAR > 250 mg/dL (level 2 hyperglycemia, measured as the percentage of readings and time spent above this level), and CV (a measure reflecting glycemic variability). These metrics were carefully analyzed to gain insights into glycemic control and fluctuations.

The insulin pumps used by participants were Medtronic MiniMed 640G and MiniMed 740G as PLGS systems, and Medtronic MiniMed 780G as the HCL system. The CGMSs used during the study period were Medtronic Guardian Sensors 3 and 4.

To obtain the parameters regarding TIR, TITR, TBR, TAR, and CV, participants’ reports were downloaded from the CareLink System Software (EMEA SOF 2400034 version). The report downloaded was titled “Assessment and progress”, and the period selected was the 90 days preceding the baseline and follow-up visit. Some participants who were not using Medtronic CGMSs prior to the study had access to specific platforms like Dexcom Clarity and LibreView, from which data from the 90 days leading up to the baseline were downloaded.

### 2.3. Statistical Analysis

Statistical analysis was performed using SPSS version 29.0.1.0. All tested parameters’ distributions were assessed for normality using the Shapiro–Wilk test. The following data were normally distributed and reported as means with standard deviation: age at baseline, diabetes duration, follow-up duration, height, HbA1c, TIR, TBR, TAR, CV, HDL-cholesterol, AST, and fT4. In contrast, the following data did not follow a normal distribution and were reported as medians accompanied by the interquartile range (IQR): weight, BMI, insulin total daily dose, insulin dose per kg of body weight, LDL-cholesterol, total cholesterol, triglycerides, ALT, TSH, anti-TPO antibodies, anti-Tg antibodies, tTg-IgA, and uACR. For categorical variables, data are presented as frequencies and percentages. Data were presented as means with standard deviations and medians with IQR to compare the parameters reflecting glycemic control at baseline and follow-up. Comparisons were performed using the paired samples t-test or the Wilcoxon signed-rank test. A 2-sided *p*-value less than 0.05 was considered statistically significant. Only individuals with baseline and follow-up data were retained when individual variables were analyzed.

### 2.4. Outcomes

The primary endpoint was to assess the difference in HbA1c levels after using the PLGS/HCL pump, and these data were available for all participants. Secondary outcomes included various measures: the percentage of time spent within the target glucose range (70 to 180 mg/dL), the percentage of time spent in the tighter target glucose range (70 to 140 mg/dL), and the percentages of time spent in hyperglycemia (categorized as >180 mg/dL, 180–250 mg/dL, and >250 mg/dL) as well as in hypoglycemia (categorized as <70 mg/dL, 54–70 mg/dL, and <54 mg/dL). The CV was also assessed, but these secondary outcomes were only evaluated in a subgroup of 30 subjects due to missing information in some individuals. Additionally, children in the study were also evaluated for diabetic complications and other autoimmune disorders, considering the prevalence and significance of these conditions among individuals with T1D.

## 3. Results

### 3.1. General Characteristics

The study encompassed a total of 51 children, primarily female (58.8%), with an average age of 10.3 ± 3.7 years. The mean height was 142.9 ± 23.3 cm, and the median weight was 35.5 kg (IQR 23.9–53). BMI had a median value of 17.3 kg/m^2^ (IQR 15.4–19.5). In terms of pubertal Tanner stages, the following distribution was observed among the participants: twenty-eight individuals (54.9%) were classified as prepubertal, three children (5.9%) were at Tanner stage 2, nine participants (17.6%) were at stage 3, four individuals (7.8%) were at stage 4, and seven individuals (13.7%) were at Tanner stage 5. The total daily insulin dose had a median of 25.5 IU (IQR 16.2–49), and the median insulin dose per kg of body weight was 0.7 IU/kg (IQR 0.6–0.9). Out of the total participants, the majority (26 children) initiated treatment using the PLGS insulin pump, while the remaining 25 opted for the HCL insulin pump. Before utilizing a pump integrated with a CGMS, 37.3% of individuals relied on a basic insulin pump, and the remaining 62.7% administered their insulin through MDI. At baseline, all children were found to be using a CGMS. The mean duration of the follow-up period was 13.2 ± 4.5 months.

The baseline characteristics of participants who entered the study are listed in Table 1.

### 3.2. Glycemic Control Parameters

Upon assessing follow-up data, all parameters under observation exhibited significant improvement. The average HbA1c level saw a considerable reduction (from 6.9 ± 0.7% to 6.7 ± 0.6%, *p* = 0.023). The TIR witnessed a significant increase (from 69.3 ± 11.2% to 76 ± 9.9%, *p* < 0.001), paralleled by a marked decline in both TBR < 70 mg/dL, (from 5.6 ± 2.9% to 3.5 ± 1.9%, *p* < 0.001) and TAR > 180 mg/dL (from 25 ± 11.2% to 20.4 ± 9.4%, *p* = 0.001). The TITR showed improvement too, rising from 47.4% ± 10.9% to 53.7% ± 10.7%, with a statistically significant result (*p* < 0.001). The CV also underwent a substantial reduction (from 37.9 ± 4.8% to 35.6 ± 4.6%, *p* = 0.001).

When comparing subintervals of TBR and TAR, significant differences were observed between the baseline and follow-up visits. The percentage of TBR for values between 54–70 mg/dL decreased from 4.3 ± 1.8 to 2.9 ± 1.5 (*p* < 0.001). The percentage of TBR for values below 54 mg/dL dropped from 1.5 ± 1.4 to 0.6 ± 0.7 (*p* < 0.001). Additionally, the percentage of TAR for values between 180–250 mg/dL decreased from 19.1 ± 6.8 to 16 ± 5.8 (*p* < 0.001), while the percentage of TAR for values above 250 mg/dL decreased from 5.9 ± 5.9 to 4.3 ± 4 (*p* = 0.037).

Most individuals (70.5%) successfully reached an HbA1c level of ≤7%. Notably, all children who reported an HbA1c level greater than 7% at the follow-up stage were using an LPGS. Moreover, it was found that the mean HbA1c at the follow-up stage was significantly lower among children utilizing HCL compared to those using LPGS (6.4 vs. 7%, *p* = 0.005).

TIR follow-up reports were available for 37 children. An impressive 72.9% of these individuals successfully achieved a TIR > 70%, and 70.2% of them obtained a TITR ≥ 50%. Interestingly, all individuals who could not achieve a TIR > 70% at the follow-up stage, except for one person, were using an LPGS insulin pump.

At the follow-up visit, there were no significant changes in median BMI (17.3 vs. 17.7 kg/m^2^, *p* = 0.4) and insulin dose per kg of body weight/day (0.72 vs. 0.75 UI/kg, *p* = 0.8).

The results regarding glycemic control parameters are summarized in Table 2.

### 3.3. Safety

Importantly, throughout the duration of the study, there were no recorded instances of severe hypoglycemia, DKA, or unscheduled hospital admission due to hyper- or hypoglycemia.

### 3.4. Complications and Associated Pathologies

A substantial fraction of participants, 29.4% (15 children), suffered from one additional autoimmune disorder: 23.5% (12 children) were diagnosed with thyroid autoimmune pathologies, and 5.8% (3 individuals) were diagnosed with celiac disease. Thyroid pathologies were represented by ten cases of Hashimoto disease and two cases of Basedow–Graves’ disease. The two children with Basedow–Graves’ were diagnosed before the baseline visit and were already under treatment with antithyroid drugs. Among the ten cases with Hashimoto disease, the diagnosis was established based on increased levels of anti-TPO antibodies in eight children, while the other two had increased levels of both anti-TPO and anti-Tg antibodies. In the case of the three children diagnosed with celiac disease, two had their diagnosis confirmed prior to this research study and were already adhering to a gluten-free diet. One child, however, received their diagnosis following the screening conducted as part of the current study. 

Of the children assessed, 38 (74.5%) had a normal BMI, falling between the 5th and 85th percentiles. Additionally, three children (7.8%) were classified as underweight, meaning they were below the 5th percentile. There were also five children (9.8%) identified as overweight, which is defined as having a BMI between the 85th and 94th percentiles, and four children (7.8%) were categorized as having obesity, with a BMI of 95th percentile or higher.

There were no individuals with raised levels of transaminases or high blood pressure identified. There were six children with abnormal lipid profiles: five with increased total and LDL-cholesterol levels and one with hypertriglyceridemia.

One person had a uACR above the normal limit of 30 mg/g. None of the participants developed diabetic retinopathy until the moment of examination and no individual presented symptoms suggesting diabetic neuropathy.

The laboratory investigations are presented in Table 1.

## 4. Discussion

Our investigation yielded compelling evidence substantiating the efficacy of both PLGS and HCL insulin pumps in optimizing glucose control amongst a pediatric population diagnosed with T1D. It is noteworthy that the application of these advanced technologies facilitated a robust improvement in all observed metrics, namely, HbA1c, TIR, TBR, TAR, and CV.

Significantly, we observed a profound reduction in the average HbA1c level, highlighting the success of these interventions in achieving a more stable and near-normal blood glucose level. This finding aligns well with previous studies emphasizing the pivotal role of insulin pumps integrated with a CGMS in managing HbA1c levels. Furthermore, a substantial number of children successfully attained an HbA1c level of ≤7%, which is often hailed as a critical benchmark in diabetes management.

An essential aspect of our study’s findings was the remarkable increase in TIR, coupled with the considerable decrease in both TBR and TAR. It underscores the importance of these technologies in enhancing the quality of life for individuals by reducing instances of hypoglycemia and hyperglycemia, leading to more stable blood glucose levels throughout the day.

Recently, the concept of TITR, which represents the percentage of time spent within the target glucose range of 70–140 mg/dL, has gained significant attention. A TITR target has not yet been established, but some researchers propose that AID users should aim for a 50% treatment goal [25].

As expected, a higher percentage of individuals using HCL successfully achieved targets recommended by guidelines compared to children using PLGS systems.

Our findings were consistent with those of other studies, which reported improvements in HbA1c, TIR, TAR, TBR, and CV in children and adolescents after starting HCL therapy [9,26,27,28]. Additionally, other studies that compared groups of individuals using HCL, LPGS, or standard care found that the first two groups had lower HbA1c, higher TIR, and lower CV compared to the third group [29,30,31,32,33]. One more study comparing HCL, LPGS, and basic insulin pump groups demonstrated that HCL was superior to both LPGS and basic insulin pumps in terms of HbA1c, TIR, TAR, and CV, with no significant differences in TBR between HCL and LPGS groups [34].

In our study, we found that children achieved a higher mean TITR of 53.7% compared to 47.8% reported in another study that examined TITR in a pediatric population using AID systems [35]. However, our findings were still lower than the 60.2% reported in a different study that assessed this parameter, among others, in a group of children using HCL systems [36].

In our cohort, the HCL system appeared to outperform the PLGS system in terms of achieving lower HbA1c levels and improved TIR percentages. This hints at the possibility of the HCL system being more efficient in automatic insulin adjustments, reflecting the superiority of a fully closed-loop system over a partially closed one. However, it is worth acknowledging that both systems demonstrated a notable improvement in overall glycemic control, and each may have a role to play in personalized treatment strategies. All these effects may contribute to fewer diabetes complications, a better quality of life, and ultimately to an increased life expectancy in the T1D population [37].

Resembling other studies’ findings, there was an increased frequency of association between T1D and other autoimmune disorders, especially thyroid pathology [38,39]. These findings emphasize and reconfirm the necessity to periodically screen for these conditions.

As strength points, we would like to highlight the longitudinal design and the use of real-world data. Additionally, the protocol excluded children who were not using a CGM at baseline, taking into consideration the importance and impact of this medical device on HbA1c. Partial remission phase influence was minimized by excluding children who had a diabetes duration of less than 6 months at baseline. A key focus of our study was the assessment of TITR, a newly introduced CGM parameter that effectively captures the time spent in euglycemia. We must also note the lack of any severe adverse events, such as severe hypoglycemia, diabetic ketoacidosis, or hospital admissions during the study period. This is an encouraging finding that underscores the safety of these technologies, alongside their demonstrated efficacy.

As weak points, this study was performed in a single center, with a limited number of people. In our country, only a few individuals benefit from PLGS systems and even fewer have access to HCL systems, hence the small number of individuals. The follow-up period was heterogeneous because of the COVID-19 pandemic and restrictions applied at that time when some individuals had to delay the follow-up visit. CGM metrics were available in a limited number of participants both at baseline and at follow-up due to limitations in accessing the relevant user data. The quality of life was not assessed in this study, as the completed questionnaires were only available at both baseline and follow-up visits in a limited number of cases. Nonetheless, achieving better glycemic control, as participants in our study did, is important for significantly enhancing quality of life and effectively reducing alarm fatigue.

## 5. Conclusions

In conclusion, our study demonstrates that both PLGS and HCL insulin pumps can significantly improve glycemic control in a pediatric population with T1D. This improvement enhances their quality of life and may help reduce the long-term complications associated with the condition. The choice of insulin delivery system should be tailored to meet each person’s individual needs and circumstances.

Additionally, our findings highlight the importance of regular screening for other autoimmune disorders in individuals with T1D. This proactive approach allows for early intervention, if necessary, and supports normal physical and neurocognitive development in these children.

## Figures and Tables

**Table 1:** Baseline characteristics of children with type 1 diabetes.

		Normal Values
Age (years)	10.3 ± 3.7	
Diabetes duration (years)	3.9 ± 2.7	
Gender		
Females, %	58.8%	
Males, %	41.2%	
BMI (kg/m^2^)	17.3 (15.4–19.5)	
Total daily dose	25.5 (16.2–49)	
Insulin dose/kg of body weight (IU/kg)	0.7 (0.6–0.9)	
Insulin regimen before HCL/PLGS		
MDI	32 (62.7%)	
Non-automated insulin pump	19 (37.3%)	
Laboratory parameter		
Total cholesterol (mg/dL)	172 (149–184)	140–200
HDL cholesterol (mg/dL)	61.8 ± 13	>40
LDL cholesterol (mg/dL)	98 (83–112)	<130
Triglycerides (mg/dL)	51 (45–71)	35–150
ALT (U/L)	14 (11–17)	8–25
AST (U/L)	22.1 ± 5.9	18–36
TSH (μIU/mL)	2.3 (1.7–2.9)	0.4–4
fT4 (ng/dL)	1.1 ± 0.1	0.8–1.8
Anti-TPO (IU/mL)	10 (3–22)	<35
Anti-Tg (IU/mL)	11 (10–13)	<115
tTg-IgA (IU/mL)	0.6 (0.1–1.3)	<10
uACR (mg/g)	7.2 (5.2–10.7)	<30

Data are expressed as mean ± standard deviation or median (25–75th percentiles) or n (%); BMI: body mass index, MDI: multiple daily injections, ALT: alanine transaminase; AST: aspartate aminotransferase; TSH: thyroid stimulating hormone; fT4: free thyroxine; anti-TPO: anti-thyroid peroxidase antibodies; anti-Tg: anti-thyroglobulin antibodies; tTg-IgA: tissue transglutaminase IgA; uACR: urine albumin–creatinine ratio.

**Table 2:** Baseline and follow-up glycemic control parameters of children with type 1 diabetes.

	Baseline	Follow-Up	*p*-Value
Primary outcome (*N*= 51)			
HbA1c (%)	6.9 ± 0.7%	6.7 ± 0.6%	*p* = 0.023
	6.9 (6.4–7.4)	6.7 (6.3–7.2)	
Secondary outcomes (*n* = 30)			
TIR (%)	69.3 ± 11.2	76 ± 9.9	*p* < 0.001
	69 (61.75–76.5)	78 (67.75–85)	
TITR (%)	47.4 ± 10.9	53.7 ± 10.7	*p* < 0.001
	45 (39–57)	55 (42–63.25)	
TBR < 70 mg/dL (%)	5.6 ± 2.9	3.5 ± 1.9	*p* < 0.001
	5 (3.75–7.25)	4 (2–5)	
TBR 54–70 mg/dL (%)	4.3 ± 1.8	2.9 ± 1.5	*p* < 0.001
	4 (3–5.25)	3 (2–3.25)	
TBR < 54 mg/dL (%)	1.5 ± 1.4	0.6 ± 0.7	*p* < 0.001 ^a^
	1 (0–2.25)	1 (0–1)	
TAR > 180 mg/dL (%)	25 ± 11.2	20.4 ± 9.4	*p* = 0.001
	26 (13.75–31.25)	19 (13–29)	
TAR 180–250 mg/dL (%)	19.1 ± 6.8	16 ± 5.8	*p* < 0.001
	21.5 (12.75–24)	15.5 (11.75–21)	
TAR > 250 mg/dL (%)	5.9 ± 5.9	4.3 ± 4	*p*= 0.037 ^a^
	4 (2–7.25)	3 (1–7.25)	
CV (%)	37.9 ± 4.8	35.6 ± 4.6	*p* = 0.001
	38.75 (33.37–40.6)	36.15 (31.82–38.32)	

HbA1c: glycated hemoglobin; TIR: time in range; TITR: time in tight range; TBR: time below range; TAR: time above range; CV: coefficient of variation. Cell contents are mean  ±  SD at the upper site and median (25–75th percentiles) at the lower site. a: Wilcoxon signed-rank test.

## Data Availability

The raw data can be obtained on request from the corresponding author.

## References

[1] Gregory G.A., Robinson T.I.G., Linklater S.E., Wang F., Colagiuri S., de Beaufort C., Donaghue K.C., Magliano D.J., Maniam J., Orchard T.J. (2022). Global incidence, prevalence, and mortality of type 1 diabetes in 2021 with projection to 2040: A modelling study. Lancet Diabetes Endocrinol..

[2] Theofilou P., Vlastos D.D. (2023). The Psychological Burden of Families with Diabetic Children: A Literature Review Focusing on Quality of Life and Stress. Children.

[3] Zahid M., Dowlatshahi S., Kansara A.H., Sadhu A.R. (2023). The Evolution of Diabetes Technology—Options Toward Personalized Care. Endocr. Pract..

[4] Kesavadev J., Saboo B., Krishna M.B., Krishnan G. (2020). Evolution of Insulin Delivery Devices: From Syringes, Pens, and Pumps to DIY Artificial Pancreas. Diabetes Ther..

[5] Medtronic. (21 April 2023). *MiniMed^TM^ 780G System*. Medtronic Diabetes. https://www.medtronicdiabetes.com/products/minimed-780g-insulin-pump-system.

[6] Jancev M., Snoek F.J., Frederix G.W.J., Knottnerus H., Blauw H., Witkop M., Moons K.G.M., van Bon A.C., DeVries J.H., Serné E.H. (2023). Dual hormone fully closed loop in type 1 diabetes: A randomised trial in the Netherlands—Study protocol. BMJ Open.

[7] Peacock S., Frizelle I., Hussain S. (2023). A Systematic Review of Commercial Hybrid Closed-Loop Automated Insulin Delivery Systems. Diabetes Ther..

[8] Leelarathna L., Choudhary P., Wilmot E.G., Lumb A., Street T., Kar P., Ng S.M. (2020). Hybrid closed-loop therapy: Where are we in 2021?. Diabetes, Obes. Metab..

[9] Da Silva J., Lepore G., Battelino T., Arrieta A., Castañeda J., Grossman B., Shin J., Cohen O. (2022). Real-World Performance of the MiniMed™ 780G System: First Report of Outcomes from 4120 Users. Diabetes Technol. Ther..

[10] Grosman B., Roy A., Lintereur L., Turksoy K., Benedetti A., Cordero T.L., Vigersky R.A., McVean J., Rhinehart A.S., Cohen O. (2024). A Peek Under the Hood: Explaining the MiniMed™ 780G Algorithm with Meal Detection Technology. Diabetes Technol. Ther..

[11] (2023). Tandem Mobi System: Small, Powerful Automated Insulin Delivery.

[12] Omnipod®5 for Healthcare Provider | Omnipod. https://www.omnipod.com/hcp/products/omnipod5.

[13] Benhamou P., Lablanche S., Vambergue A., Doron M., Franc S., Charpentier G. (2020). Patients with highly unstable type 1 diabetes eligible for islet transplantation can be managed with a closed-loop insulin delivery system: A series of N-of-1 randomized controlled trials. Diabetes Obes. Metab..

[14] Templer S. (2022). Closed-Loop Insulin Delivery Systems: Past, Present, and Future Directions. Front. Endocrinol..

[15] FDA FDA Clears New Insulin Pump and Algorithm-Based Software to Support Enhanced Automatic Insulin Delivery.

[16] Bionic Pancreas Research Group (2022). Multicenter, Randomized Trial of a Bionic Pancreas in Type 1 Diabetes. N. Engl. J. Med..

[17] ElSayed N.A., Aleppo G., Bannuru R.R., Bruemmer D., Collins B.S., Ekhlaspour L., Hilliard M.E., Johnson E.L., Khunti K., American Diabetes Association Professional Practice Committee (2023). 14. Children and Adolescents: Standards of Care in Diabetes—2024. Diabetes Care.

[18] de Bock M., Codner E., Craig M.E., Huynh T., Maahs D.M., Mahmud F.H., Marcovecchio L., DiMeglio L.A. (2022). ISPAD Clinical Practice Consensus Guidelines 2022: Glycemic targets and glucose monitoring for children, adolescents, and young people with diabetes. Pediatr. Diabetes.

[19] Wright L.A.-C., Hirsch I.B. (2017). Metrics Beyond Hemoglobin A1C in Diabetes Management: Time in Range, Hypoglycemia, and Other Parameters. Diabetes Technol. Ther..

[20] Danne T., Nimri R., Battelino T., Bergenstal R.M., Close K.L., DeVries J.H., Garg S., Heinemann L., Hirsch I., Amiel S.A. (2017). International Consensus on Use of Continuous Glucose Monitoring. Diabetes Care.

[21] Battelino T., Danne T., Bergenstal R.M., Amiel S.A., Beck R., Biester T., Bosi E., Buckingham B.A., Cefalu W.T., Close K.L. (2019). Clinical Targets for Continuous Glucose Monitoring Data Interpretation: Recommendations from the International Consensus on Time in Range. Diabetes Care.

[22] ElSayed N.A., Aleppo G., Bannuru R.R., Beverly E.A., Bruemmer D., Collins B.S., Cusi K., Darville A., Das S.R., American Diabetes Association Professional Practice Committee (2023). Summary of Revisions: Standards of Care in Diabetes—2024. Diabetes Care.

[23] ElSayed N.A., Aleppo G., Bannuru R.R., Bruemmer D., Collins B.S., Ekhlaspour L., Hilliard M.E., Johnson E.L., Khunti K., American Diabetes Association Professional Practice Committee (2023). 6. Glycemic Goals and Hypoglycemia: Standards of Care in Diabetes—2024. Diabetes Care.

[24] ElSayed N.A., Aleppo G., Bannuru R.R., Bruemmer D., Collins B.S., Ekhlaspour L., Hilliard M.E., Johnson E.L., Khunti K., American Diabetes Association Professional Practice Committee (2023). 1. Improving Care and Promoting Health in Populations: Standards of Care in Diabetes—2024. Diabetes Care.

[25] Castañeda J., Arrieta A., Heuvel T.v.D., Battelino T., Cohen O. (2023). Time in Tight Glucose Range in Type 1 Diabetes: Predictive Factors and Achievable Targets in Real-World Users of the MiniMed 780G System. Diabetes Care.

[26] Garg S.K., Weinzimer S.A., Tamborlane W.V., Buckingham B.A., Bode B.W., Bailey T.S., Brazg R.L., Ilany J., Slover R.H., Anderson S.M. (2017). Glucose Outcomes with the In-Home Use of a Hybrid Closed-Loop Insulin Delivery System in Adolescents and Adults with Type 1 Diabetes. Diabetes Technol. Ther..

[27] Forlenza G.P., Pinhas-Hamiel O., Liljenquist D.R., Shulman D.I., Bailey T.S., Bode B.W., Wood M.A., Buckingham B.A., Kaiserman K.B., Shin J. (2019). Safety Evaluation of the MiniMed 670G System in Children 7–13 Years of Age with Type 1 Diabetes. Diabetes Technol. Ther..

[28] Petrovski G., Al Khalaf F., Campbell J., Umer F., Almajaly D., Hamdan M., Hussain K. (2020). One-year experience of hybrid closed-loop system in children and adolescents with type 1 diabetes previously treated with multiple daily injections: Drivers to successful outcomes. Acta Diabetol..

[29] Ng S.M., Wright N.P., Yardley D., Campbell F., Randell T., Trevelyan N., Ghatak A., Hindmarsh P.C. (2023). Real world use of hybrid-closed loop in children and young people with type 1 diabetes mellitus—A National Health Service pilot initiative in England. Diabet. Med..

[30] Lunati M.E., Morpurgo P.S., Rossi A., Gandolfi A., Cogliati I., Bolla A.M., Plebani L., Vallone L., Montefusco L., Pastore I. (2022). Hybrid Close-Loop Systems Versus Predictive Low-Glucose Suspend and Sensor-Augmented Pump Therapy in Patients with Type 1 Diabetes: A Single-Center Cohort Study. Front. Endocrinol..

[31] Wadwa R.P., Reed Z.W., Buckingham B.A., DeBoer M.D., Ekhlaspour L., Forlenza G.P., Schoelwer M., Lum J., Kollman C., Beck R.W. (2023). Trial of Hybrid Closed-Loop Control in Young Children with Type 1 Diabetes. N. Engl. J. Med..

[32] Boughton C.K., Allen J.M., Ware J., Wilinska M.E., Hartnell S., Thankamony A., Randell T., Ghatak A., Besser R.E., Elleri D. (2022). Closed-Loop Therapy and Preservation of C-Peptide Secretion in Type 1 Diabetes. N. Engl. J. Med..

[33] Ware J., Boughton C.K., Allen J.M., Wilinska M.E., Tauschmann M., Denvir L., Thankamony A., Campbell F.M., Wadwa R.P., Buckingham B.A. (2022). Cambridge hybrid closed-loop algorithm in children and adolescents with type 1 diabetes: A multicentre 6-month randomised controlled trial. Lancet Digit. Health.

[34] Bombaci B., Passanisi S., Alibrandi A., D’arrigo G., Patroniti S., Averna S., Salzano G., Lombardo F. (2022). One-Year Real-World Study on Comparison among Different Continuous Subcutaneous Insulin Infusion Devices for the Management of Pediatric Patients with Type 1 Diabetes: The Supremacy of Hybrid Closed-Loop Systems. Int. J. Environ. Res. Public Health.

[35] Piona C., Passanisi S., Bombaci B., Marigliano M., Lombardo F., Mancioppi V., Morandi A., Maffeis C., Salzano G. (2024). ISPED Diabetes Study Group Time in tight range in automated insulin delivery system users: Real-world data from children and adolescents with type 1 diabetes. Diabetes Obes. Metab..

[36] Eviz E., Killi N.E., Karakus K.E., Can E., Gokce T., Mutlu G.Y., Hatun S. (2024). Assessing the feasibility of time in tight range (TITR) targets with advanced hybrid closed loop (AHCL) use in children and adolescents: A single-centre real-world study. Diabet. Med..

[37] Ioacara S., Sava E., Georgescu O., Sirbu A., Fica S. (2018). Recent diabetes-related mortality trends in Romania. Acta Diabetol..

[38] Popoviciu M.S., Kaka N., Sethi Y., Patel N., Chopra H., Cavalu S. (2023). Type 1 Diabetes Mellitus and Autoimmune Diseases: A Critical Review of the Association and the Application of Personalized Medicine. J. Pers. Med..

[39] Mäkimattila S., Harjutsalo V., Forsblom C., Groop P.-H. (2020). Every Fifth Individual with Type 1 Diabetes Suffers From an Additional Autoimmune Disease: A Finnish Nationwide Study. Diabetes Care.

